# Incidence and risk factors for central venous catheter-related thrombosis in hematological patients

**DOI:** 10.1007/s12032-013-0772-8

**Published:** 2013-11-29

**Authors:** Monika Joks, Anna Czyż, Dariusz Popławski, Mieczysław Komarnicki

**Affiliations:** 1 Department of Hematology, Poznan University of Medical Sciences, Szamarzewskiego 84, 61-569 Poznan, Poland; 2 Department of Radiology, Poznan University of Medical Sciences, Szamarzewskiego 84, 61-569 Poznan, Poland

**Keywords:** Catheter-related thrombosis, Exit-site infection, Blood stream infection, Doppler ultrasound

## Abstract

Catheter-related thrombosis (CRT) is a serious complication in hematological patients, but the risk factors for its occurrence are not well established. The study objectives were to estimate the incidence of CRT and to identify the risk factors for developing CRT in hematological patients. In a prospective setting, 104 consecutive patients with 200 insertions of central venous catheters were enrolled into the study. The patients were screened for CRT by compression Doppler ultrasound every 10–14 days. Additionally, ultrasonography was performed in the case of clinical symptoms suggesting CRT. Over the course of 6,098 catheter days of follow-up, the incidence of CRT was 13.5 %. In 18/27 cases (66.6 %), radiological evidence of CRT was preceded by clinical symptoms. However, in 9/27 (33.3 %), CRT was clinically asymptomatic. The median times to symptomatic and asymptomatic CRT were 17 (range 1–49) and 8 (range 1–16) catheter days, respectively. In univariate analysis, the risk factors for CRT were exit-site infection (ESI) (*P* < 0.001), two or more prior chemotherapy lines (*P* = 0.015), catheter-related blood stream infection (*P* < 0.001), and Coagulase-negative staphylococci infection (*P* = 0.002). In multivariate analysis, ESI (OR 5.0; 95 % CI 1.6–6.3; *P* = 0.006) and two or more prior chemotherapy lines (OR 3.57; 95 % CI 1.27–10.11; *P* = 0.015) remained significantly associated with the risk of CRT. The results of our study provide information regarding the characteristic features of the patients who are at high risk of thrombosis, for whom Doppler ultrasound screening should be considered.

## Introduction

Central venous catheters (CVCs) are frequently used in hematological patients in order to administer chemotherapy and other infusates, as well as blood and stem cell products. In addition, CVCs may also be used for blood sampling. Reported complications consist of mechanically related ones, CVC-related infections, and thrombosis. The pathogenetic mechanisms of catheter-related thrombosis (CRT) include the intravascular foreign surface, obstruction of the venous flow, and trauma to the venous wall. Although the pathogenesis of CRT is multifactorial, venous endothelial damage appears to play the most important role. This damage may be the result of mechanical irritation by the line [1], or may arise as a consequence of the use of certain drugs, e.g., cytostatics, antibiotics, and of parenteral nutrition. Moreover, there is accumulating evidence that CRT and CVC-related infections are not separate entities, but seem to have a bidirectional relationship [2–7].

Previous studies of CRT have mainly been based on retrospective analyses of oncological and intensive care unit patients. There is a little data available relating to hematological patients. These patients differ from others with respect to more severe and prolonged thrombocytopenia and leucopenia, as well as to abnormalities of plasma coagulation. Hematological patients are usually treated by the implantation of short-term non-tunneled percutaneous catheters inserted via the internal jugular, subclavian, or femoral veins.

There are discrepancies in the literature relating to CRT. Many risk factors have been postulated, including the type of catheter, insertion site, CVC tip localization, previous CRT, method of treatment, CVC-related infection, difficulties during insertion, and inherited thrombophilia [8–13].

According to the literature, CRT is clinically silent in two-thirds of all CRT cases. In these cases, the clot does not close the vein completely [14]. Among hematological patients, clinically asymptomatic CRT is diagnosed in 1.5–34 % of them [8, 15, 16]. In these patients, CRT is usually diagnosed after 4–20 days, on average within 11 days [2]. The clinical symptoms of CRT vary widely and consist of swelling or pain, numbness, erythema of the extremity, phlegmasia, and venous distension. Symptomatic CRT occurs in 1.2–13 % of hematological patients [2, 3, 16–21].

The diagnosis of CRT by venography is considered as the “gold standard,” but it has some important limitations. This invasive method requires the administration of contrast media and radiography and is associated with the risk of serious complications. For these reasons, venography has been largely replaced by compression Doppler ultrasound in many centers for the diagnosis of CRT. The advantages of this method include its wide availability, noninvasiveness, and relatively short duration. The sensitivity of Doppler ultrasound ranges from 78 to 100 % and the specificity from 82 to 100 % [22].

In our study, the aims were to estimate the incidence of CRT and to identify the risk factors for developing this condition in hematological patients.

## Materials and methods

### Study population

The study was performed in the Department of Hematology, Poznan University of Medical Sciences.

A registry of patients undergoing CVC insertion for therapy was initiated in March 2008 and completed in March 2010. Consecutive patients who underwent CVC positioning were eligible to this study, and the only one exclusion criterion was the lack of consent to participate. The study was approved by the Ethics Committee of the University. Informed consent was obtained from all patients.

### Procedures of catheter insertion and maintenance

Catheter insertions were performed in a surgery by a staff physician under standard sterile conditions—sterile gloves and small drape. Before the procedure, the patients took an antiseptic shower. At the time of catheter insertion, the insertion site was cleaned with 1 % alcohol-based povidone-iodine solution.

The same type of central venous catheter, a 16-cm-long, 3-lumen polyurethane catheter impregnated with chlorhexidine/silver sulfadiazine (Arrow) was used for every patient.

The decision on the choice of insertion site was taken by the responsible physician and was mainly based on personal preferences and the patient’s underlying characteristics. In patients with a history of previous CRT, the CVC was inserted in another vein or into the same vein after exclusion of CRT.

CVC insertion was performed under local anesthesia by the percutaneous technique, guided by anatomic landmarks.

In those patients in whom the CVC had been inserted in the internal jugular or subclavian veins, location of the tip of the catheter tip in the superior vena cava or right atrium was confirmed by standard chest radiography (Cosmos Siemens).

Neither antibiotic prophylaxis nor any thromboprophylaxis was administered. Maintenance procedures were performed by trained hematological nurses and included the replacement of sterile gauze dressings at least every 48 h, or earlier after contamination or detachment. Where transparent polyurethane dressings (Tegaderm Film 3 M) were need, these changes were made not less frequently than weekly. During the exchange, the CVC site was disinfected by Skinsept preparation (alcohol-based and hydrogen peroxide solution). Moreover, all catheter’s lumens were flushed with 0.9 % NaCl daily.

### Follow-up period

The observation time covered the period from insertion to removal of the catheter. The catheter was removed at the end of hospitalization, or earlier in cases of mechanical obstruction, and in some cases of CRT and/or catheter-related blood stream infection (CRBSI) and/or exit-site infection (ESI).

### Doppler ultrasound

Ultrasound with Doppler and color imaging (GE Voluson 730 Pro) was performed every 10–14 days and additionally if any clinical symptoms suggesting CRT were noted.

Every evaluation was carried out by the same radiologist. CRT was diagnosed after finding a thrombus with partial or total occlusion of the vessel. In cases of internal jugular or femoral vein thrombosis, additional confirmation was provided by a positive compression test.

#### Microbiology surveillance

The patients were screened for CVC-related infections. Two entities were distinguished: ESI and CRBSI. The CVC-related infections were defined according to the Clinical Practice Guidelines for the Diagnosis and Management of Intravascular Catheter-Related Infections: 2009 Update by the Infectious Diseases Society of America (IDSA) [23].

At each episode of fever onset (body temperature >38.2° C) or other symptoms of infection, paired qualitative blood cultures were performed from both the catheter and a peripheral vein. The blood samples were injected into BacT/Alert FN and BacT/Alert FA culture media. Analyses were performed using a computerized system for monitoring blood cultures (BacT/Alert 3D Bio-Merieux). The differential time to positivity (DTP) was evaluated. DTP was defined according to the IDSA (23) as a growth in a blood culture obtained through a catheter hub and detected by an automated blood culture system, at least 2 h earlier than a culture of simultaneously drawn peripheral blood of equal volume.

Additionally, swab cultures (Eurotubo Collection Swab, Delta Lab) were taken in the presence of clinical signs of ESI with exudation. Catheter distal tip culture was performed routinely.

The identification and drug sensitivity of cultured microorganisms were made by the Vitek 2 Compact system (Bio-Merieux) with standard interpretation of susceptibility according to the Clinical and Laboratory Standards Institute (CLSI). For some pathogens, sensitivity was estimated using the E-test.

### Laboratory data

Platelet cell count (hematology analyzer Abbott Cell Dyn 3700) was evaluated in patients who developed CRT. The plasma D-Dimers concentration (ACL Elite Pro, commercial test kit Instrumentation Laboratory/Comesa) was analyzed in all patients.

### Statistical analysis

The Pearson chi-square statistical test and Fisher–Freeman–Halton 2-sided test were used to compare the general characteristics of patients with and without CRT. Univariate analysis of CRT for each potential risk factor was performed using logistic regression. Exposures for which the *P* value was <0.10 in the univariate analysis were submitted to a multivariate conditional logistic regression model. Backward stepwise regression procedures were used to develop the final multivariate model. A *P* value <0.05 was considered as significant. Odds ratios (ORs) and 95 % CIs (confidence intervals) were calculated on the basis of the final model.

All statistical analyses were performed using Statistica for Windows (StatSoft, Inc. 2001).

## Results

### Patients and CVC positioning

A total of 200 cases of CVC positioning in 104 patients with a median age of 45 years (range 18–76) were enrolled in the study.

The main patient and catheter characteristics are presented in Table 1.

**Table 1:** Patient and catheter characteristics

Characteristics	Number (%)
Sex	
Male	118 (59)
Female	82 (41)
Diagnosis	
Acute myeloblastic leukemia	80 (40)
Acute lymphoblastic leukemia	37 (18.5)
B cell chronic lymphocytic leukemia	10 (5)
Non-Hodgkin lymphoma	44 (22)
Hodgkin lymphoma	17 (8.5)
Other hematological disorders	4 (2)
Disease status	
Complete/partial remission	148 (74)
Number of prior chemotherapy lines	
0	102 (51)
1	69 (34.5)
2	26 (13)
3	2 (1)
4	1 (0.5)
Treatment data	
Prolonged chemotherapy infusion (>12 h)	86 (43)
Cisplatin-based polychemotherapy	41 (20.5)
Catheter placement	
Right internal jugular vein	109 (54.5)
Left internal jugular vein	41 (20.5)
Right subclavian vein	17 (8.5)
Left subclavian vein	7 (3.5)
Right femoral vein	23 (11.5)
Left femoral vein	3 (1.5)
Tip localization	
Superior vena cava	154 (77)
Superior vena cava/right atrium	12 (6)
Right atrium	7 (3.5)

The overall median follow-up time was 27 days (range 4–183), for a total of 6,098 catheter days.

The median follow-up of patients who developed CRT was 22 days (range 12–49). In 17/27 cases, the catheter was removed after confirming CRT. All the patients with CRT were treated with enoxaparin, with the therapeutic dose adjusted to the platelet count and the clinical symptoms of thrombocytopenia.

### CRT incidence

Of the total of 200 insertions, CRT was confirmed in 27 (13.5 %). Radiological evidence of CRT was preceded by clinical symptoms in 18 of these 27 cases (66.6 %). However, in 9 of these 27 patients (33 %) and in 4.5 % of the total group of 200 insertions, CRT was clinically silent.

The median times to symptomatic CRT and clinically asymptomatic CRT were 17 catheter days (range 1–49) and 8 catheter days (range 1–16), respectively. Thrombosis was confirmed within 15 days from catheter placement in 11 cases, between 15 and 30 days in 14 cases, and beyond 30 days in 2 cases. CRT was associated with CVC-related infections in 13/27 cases (48.1 %). Thirteen cases of ESI were present, and additionally, 6 cases met the criteria of CRBSI. Based on the Pearson chi-square test, we analyzed incidence of CRT in the different subgroups of patients. The incidence rate of CRT differed significantly between groups of patients stratified by a number of prior chemotherapy lines, CRBSI, exit-site infection, and infection by Coagulase-negative staphylococci (CoNS) (Table 2). In contrast, no association was found between the incidence rate of CRT and age of patient, prior CRT, underlying hematological disorder, time of cytostatic infusion (>12 vs. ≤12 h), type of chemotherapy (cisplatin-based chemotherapy vs. others), tip localization, D-dimer concentration, or catheter insertion site.

**Table 2:** Incidence of venous catheter-related thrombosis in the subgroups of patients

Groups	CRT incidence rate	*P*
Number of prior chemotherapy lines		
1 versus 2 or more	11.1 versus 27.6 %	0.033
CRBSI		
No versus yes	11.6 versus 30.0 %	0.009
Exit-site infection		
No versus yes	8.6 versus 18.0 %	<0.001
Infection by CoNS		
No versus yes	10.7 versus 34.7 %	0.001

*CRT* catheter-related thrombosis, *CRBSI* catheter-related blood stream infection, *CoNS* Coagulase-negative staphylococci

### Risk factors

Based on univariate logistic regression analysis, we identified four risk factors of CRT (Table 3).

**Table 3:** Univariate analysis of risk factors of catheter-related thrombosis

Risk factor	OR (95 % CI)	*P*
Two or more prior chemotherapy lines	3.22 (1.24–8.36)	0.015
Exit-site infection	6.05 (2.51–14.5)	<0.001
CRBSI	3.83 (1.29–11.3)	0.001
CoNS infection	4.43 (1.65–11.9)	0.002

*OR* odds ratio, *CI* confidence interval, *CoNS* coagulase-negative staphylococci, *CRBSI* catheter-related blood stream infection

Multiple logistic regression analysis revealed two independent risk factors of CRT: exit-site infection (OR 5.01; 95 % CI 1.6–6.3; *P* = 0.006) and two or more prior chemotherapy lines (OR 3.57; 95 % CI 1.27–10.11; *P* = 0.015).

## Discussion

CRT is a serious complication in hematological patients. Few studies relating to the incidence of CRT and associated risk factors in patients treated for hematological disorders have been published. Therefore, our aims were to evaluate this incidence and to identify risk factors for CRT in patients hospitalized in our center.

In all the studies reported in the literature, CRT was investigated in patients in whom the catheters were inserted via different types of vascular access port. The catheters that were of either the tunneled or non-tunneled types were impregnated or unimpregnated, placed in peripheral or central veins, and had different numbers of lumen. Our study was carried out on a large group of patients with the same type of CVC 3 lumen polyurethane catheter impregnated with chlorhexidine/silver sulfadiazine (Arrow).

We confirmed the incidence of CRT in 27 catheterization cases, representing a prevalence of 13.5 %. In almost half the cases, CRT was associated with CVC-related infections. In two-thirds of the cases, the radiological signs were preceded by clinical symptoms. In the remainder, CRT was diagnosed based by Doppler ultrasound screening. Our results are not consistent with data available in the literature, and van Rooden et al. [24] found that episodes of asymptomatic CRT occur in two-thirds of all CRT. The difference may originate from the fact that they performed Doppler ultrasound every 7 days, compared to the 10–14 days in our investigation. As a result, the incidence of asymptomatic CRT is probably underestimated in our study.

We were not able to predict the risk of symptomatic CRT on the basis of asymptomatic CRT, due to the small number of patients affected and the fact that in most cases, the treatment was implemented immediately after detecting the thrombosis. According to the literature, clinically silent CRT seems to be relevant, because it increases the risk of developing symptomatic thrombosis sevenfold, compared with negative Doppler ultrasound findings [24]. The guidelines for screening Doppler ultrasound testing in hematological patients have not yet been established. Based on the fact that a significant percentage of clinically silent CRT evolves into symptomatic CRT, we believe strongly that Doppler ultrasound screening should be considered in this group of patients. In our study, the median time from the last normal Doppler ultrasound to the Doppler ultrasound with CRT was 8 days (range 1–16). Based on these data, Doppler ultrasound screening for CRT every seven days seems to be advisable, especially in patients with a high risk of developing CRT.

In our group of 200 catheter insertions, the median time to diagnosis of CRT (both symptomatic and asymptomatic) was 17 days. Noteworthy is the fact that the longest time to CRT after catheter insertion, beyond 30 days occurred in only two cases. These data are consistent with reports in the literature [2]. The treatment of hematological patients is often complicated by grade 3–4 thrombocytopenia, which might be expected to reduce the risk of CRT. However, this presumption is not reflected in data in the literature [8]. In the presence of inflammation, inflammatory mediators stimulate the formation of young and more thrombogenic platelets. Furthermore, inflammatory mediators release extremely large multimers of von Willebrand factor from the endothelium, which stimulates the formation of large platelet thrombi [25]. In our CRT patients, the median platelet count (PLT) was 45 (range 10–592) G/L. These findings are justified in the context of described mechanisms of increased thrombotic risk during infection, irrespective of concomitant thrombocytopenia.

In our study, we analyzed many parameters as potential risk factors. We found no difference in the incidence of CRT in the female group compared to the male group. With regard to age, our patients were divided patients into 4 groups: those of 18–29, 30–39, 40–49, and ≥50 years, respectively. The patients in the oldest age group suffered from many of the co-morbidities predisposing to thrombosis. Despite this, none of the groups showed an increased risk of CRT. These data are consistent with previous reports in the literature [1, 8]. In our study, there was not also a significant risk of CRT recurrence. We found two reports in the literature with the same conclusion [1, 8]. We also studied the different CVC insertion sites as a potential risk factor of CRT. We did not find an increased risk of CRT in any of the catheter placements such as the right internal jugular, the left internal jugular, the right subclavian, the left subclavian, the right femoral, or the left femoral vein. We also compared the incidence of CRT after placement in the internal jugular veins versus to subclavian or femoral veins and also compared the internal jugular and subclavian veins versus femoral veins. There were no differences. We also found no difference in the incidence of CRT with catheter placement on the right side of the body versus the left side. In summary, we did not find any correlation between the risk of CRT and the catheter placement site. There are reports that locating the catheter tip in the brachio-cephalic vein or distal part of the superior vena cava poses a greater risk of CRT than locating the tip in the proximal part of the superior vena cava or right atrium results in an increased risk of CRT [9, 10]. The results of our study do not confirm this finding.

Among the other potential risk factors, we also analyzed underlying hematological disorders, but we did not find an increased incidence of CRT in any group of patients. In the available literature, there is one report concerning a pediatric group which claimed ALL diagnosis as a risk factor for thrombosis [26], but our findings do not support this observation.

Due to the fact that endothelial cell damage triggers the initiation of thrombotic processes, the use of intensive chemotherapy may be a risk factor for thrombotic complications. In our study, we analyzed the relationship between CRT and cisplatin-based regimens. Cisplatin, one of the inorganic compounds of platinum, works independently of the phase of the cell cycle, inhibits DNA synthesis to form intra- and interchain DNA connections, and is characterized by a long half-life (20–30 h). In our group of patients, cisplatin in a dose of 25 mg/m^2^ was administered as a continuous infusion for 4 days as a part of ESHAP (etoposide, cisplatin, cytarabine, methylprednisolone) chemotherapy. There are data in the literature indicating an increased risk of deep vein thrombosis, pulmonary embolism, and ischemic stroke in cancer patients treated with cisplatin-based chemotherapy [27]. However, we were unable to find any published reports about the risks of CRT after cisplatin, and in our study, we did not find any increased risk of CRT in those of our patients treated with cisplatin-based polychemotherapy. Similarly, the results of our study do not confirm the influence of prolonged infusions (>12 h) of other cytotoxic drugs (cytarabine, ifosfamide, cisplatin, methotrexate) on the risk of CRT.

Treatment with subsequent lines of chemotherapy, other angiotoxic medications, and repeated CVC placements can all damage the endothelium. We therefore analyzed the risk of CRT depending on the number of prior chemotherapy lines. Comparing the incidence of CRT in patients who underwent two or more chemotherapy lines with the experience of patients previously untreated or treated with only one line of chemotherapy, we found a statistically significant increased incidence of CRT in the first group (*P* = 0.033). Univariate analysis using logistic regression confirmed this correlation between previous therapy with two or more chemotherapy lines and CRT (*P* = 0.015). Multivariate analysis confirmed treatment with more than 2 prior chemotherapy lines is an independent risk factor for CRT, increasing the risk of CRT more than threefold. Our results may be important from a practical point of view, suggesting the need for increased clinical supervision and Doppler ultrasound screening in heavily pretreated patients. So far, we are aware this relationship has not been reported previously.

The D-dimer concentration in plasma was another laboratory parameter analyzed in our study as a risk factor of CRT. We did not find any correlation between an elevated D-dimer concentration and the incidence of CRT. Because of the multiplicity of causes increasing the D-dimer concentration, the results obtained are not surprising, but clearly illustrate the fact that this concentration has no value as a diagnostic test of CRT in hematological patients.

Many studies have shown a strong relationship between the pathogenesis of infectious and thrombotic processes [2, 5, 7]. A major factor connecting CRT and CVC-related infections is the formation of a fibrin sheath around the catheter and inside the catheter lumen. CVC-related thrombi consist of many proteins, such as fibrin, fibronectin, collagen, and laminin. Microorganisms, especially Staphylococcus aureus and Staphylococcus epidermidis, easily adhere to the catheter and produce coagulase enzymes enhancing the thrombotic process. This relationship was analyzed in our study. We confirmed the presence of CRBSI in 10 % cases. Literature data on the incidence of CRBSI in hematological patients varies widely, ranging from 0 to 20.8 %. These differences arise from the different definitions used for CRBSI. In our study, 76.1 % of CRBSI cases were the result of staphylococcal infections. The most common pathogenic factor for CRBSI was Staphylococcus epidermidis MRS. This bacterium belongs to the coagulase-negative staphylococci and forms a part of the skin flora. Our findings relating to both the species of pathogen and the incidence of infection are in line with the literature data [21]. Concomitant CRT was confirmed in 33.3 % of our CRBSI cases. A preliminary analysis showed an increased incidence of CRT in patients meeting the criteria for CRBSI (*P* = 0.009). Analysis by a logistic univariate regression model confirmed the association between CRBSI and CRT (*P* < 0.001), but multivariate analysis did not show that CRBSI is an independent factor of CRT.

Due to the fact that ESI is usually the first manifestation of a catheter-related infection, we analyzed its relationship with CRT. Clinical symptoms of ESI were observed in 36 cases, with purulent drainage in 12 of them. The microorganisms cultured from purulent drainage belonged to staphylococci in more than 80 % cases. We demonstrated an increased incidence of CRT in patients with ESI (*P* < 0.001) that was observed in one-third of the ESI cases. Multivariate analysis identified ESI as an independent risk factor, increasing the risk of the CRT fivefold. On the basis of our results, we conclude that the risk of CRT correlates with the incidence of CVC-related infections, and preventive strategies should therefore include the effective prevention and treatment of CVC-related infections.

In summary, our results indicate that those hematological patients with CVC who present exit-site infection, or who were heavily pretreated, are at high risk of developing CRT. We therefore recommend the use of Doppler screening as an aid in the early diagnosis of CRT.
